# Clinical efficacy of transcutaneous tibial nerve stimulation (TTNS) versus sham therapy (part I) and TTNS versus percutaneous tibial nerve stimulation (PTNS) (part II) on the short term in children with the idiopathic overactive bladder syndrome: protocol for part I of the twofold double-blinded randomized controlled TaPaS trial

**DOI:** 10.1186/s13063-021-05117-8

**Published:** 2021-04-02

**Authors:** Lynn Ghijselings, Catherine Renson, Johan Van de Walle, Karel Everaert, Anne-Françoise Spinoit

**Affiliations:** 1 Urology Department, Ghent University Hospital, Ghent, Belgium; 2 Paediatric Nephrology Department, Ghent University Hospital, Ghent, Belgium

**Keywords:** Urinary bladder, Overactive bladder, Nocturnal enuresis, Children, Transcutaneous electric nerve stimulation, Randomized controlled trial

## Abstract

**Background:**

Transcutaneous tibial nerve stimulation (TTNS) and percutaneous tibial nerve stimulation (PTNS) are effective and safe therapies for overactive bladder (OAB) syndrome in adults. However, few randomized sham-controlled trials have been conducted in a pediatric population. To our knowledge, both therapies never have been compared in children.

**Aim:**

The aim of the complete study is twofold: (1) to assess the efficacy of TTNS therapy on bladder symptoms after 12 weeks of treatment in a pediatric population with idiopathic overactive bladder syndrome (iOAB) and/or nocturnal enuresis (part I) and (2) to assess the effect of TTNS compared to PTNS (part II). In this article, we aim to present the protocol of the first part of the TaPaS trial (TTNS, PTNS, sham therapy).

**Methods:**

Part I of the TaPaS trial is set up as a single-center randomized-controlled trial. Children, aged from 5 to 12 years with iOAB and/or nocturnal enuresis, are assigned to two groups by computer-generated randomization: TTNS therapy (intervention) and sham therapy (control). The primary outcome is the percentage difference in average voided volume (AVV) between baseline and after 12 weeks of treatment. Secondary endpoints are the percentage difference in supervoid volumes, number of urinary incontinence episodes/24 h and in voiding frequency, the difference in parent reported outcomes between baseline and after 12 weeks of treatment, and the duration of clinical response.

**Discussion:**

We hypothesize that TTNS is a non-inferior treatment for iOAB in children compared to PTNS therapy. Since literature is inconclusive about the efficacy of TTNS in a pediatric population, a sham-controlled RCT on TTNS will be conducted (part I). A protocol for a prospective randomized sham-controlled trial has been developed. Enrolment has started in November 2018. Study completion of part I is expected by August 2021.

**Trial registration:**

ClinicalTrials.gov NCT 04256876. Retrospectively registered on February 5, 2020.

**Supplementary Information:**

The online version contains supplementary material available at 10.1186/s13063-021-05117-8.

## Administrative information

The order of the items has been modified to group similar items (see http://www.equator-network.org/reporting-guidelines/spirit-2013-statement-defining-standard-protocol-items-for-clinical-trials/).

**Table:** 

Title {1}	Clinical efficacy of Transcutaneous tibial nerve stimulation (TTNS) versus Sham therapy (Part I) and TTNS versus Percutaneous tibial nerve stimulation (PTNS) (Part II) on the short term in children with the idiopathic overactive bladder syndrome (TaPaS): Protocol for Part I of a two-fold randomized controlled trial.
Trial registration {2a and 2b}.	ClinicalTrials.gov (NCT 04256876). Retrospectively registered on February 5, 2020.
Protocol version {3}	Version 2. EC2018/1267.
Funding {4}	Financial support for the employment of investigators and the purchase of equipment was delivered by a part of the grant of the OptiLUTS Chair by Medtronic (Grant number: A 1357636).
Author details {5a}	Lynn Ghijselings^1^, Catherine Renson^1^, Mieke Waterschoot^1^, Johan Van de Walle^2^, Karel Everaert^1^, Anne-Françoise Spinoit^1^. 1. Urology Department, Ghent University hospital, Ghent, Belgium. 2. Paediatric Nephrology Department, Ghent University hospital, Ghent, Belgium. Corresponding author: Lynn Ghijselings Email: lynn.ghijselings@ugent.be Tel: +32 9 33 21 353 ORCID: 0000-0001-5144-4021
Name and contact information for the trial sponsor {5b}	Ghent University Hospital Corneel Heymanslaan 10, 9000 Ghent Belgium. Tel: +32 9 332 21 11. Clinical Trials Unit E-mail: HIRUZ.CTU@UZGENT.BE Tel.: +32 9 332 05 00
Role of sponsor {5c}	- Support in the preparation of a correct and complete submission package for the Ethics Committee and Competent Authority. - Performing on-site and remote monitoring according ICH-GCP for all clinical trials. - Providing a no-fault insurance.

## Introduction

### Background and rationale {6a}

The reported prevalence of daytime lower urinary tract dysfunctions (LUTD) in children ranges widely from 1 to 20% [1]. Among these, overactive bladder syndrome (OAB) as a storage dysfunction and dysfunctional voiding as an emptying dysfunction are the two main entities. Failure to achieve continence (normally reached by the age of three to four), urgency, frequency, hesitancy, and frequent urinary tract infections (UTIs) are reported complaints. Nocturnal enuresis, without daytime LUTD, is considered as a separate entity and has an estimated prevalence of 5–10% at the age of seven [1].

In the management of both OAB and nocturnal enuresis, the objective is to increase the bladder capacity if reduced for the age. Conservative therapy like lifestyle advice and behavioral modifications, including bladder re-education and bowel management, is the first step in a multimodal approach (urotherapy). In case conservative therapy fails, anticholinergic drugs are the mainstay of medical treatment. As an alternative, in case one is reluctant towards pharmacotherapy or in case pharmacotherapy has failed, peripheral neuromodulation can be offered.

Different types of peripheral neuromodulation have been practiced in the pediatric population like transcutaneous electrical nerve stimulation (TENS)—on the sacrum or over the posterior tibial nerve (TTNS) and percutaneous tibial nerve stimulation (PTNS) [2, 3]. However, to our knowledge, no exact data on how frequently adopted this treatments are, are published.

In our department, PTNS is the alternative treatment for OAB in case there are contra-indications for the use of drug therapy or in case a non-drug therapy is preferred. It is proven to be effective and safe in children with iOAB [4]. However, the time-consuming weekly in-office visits and the percutaneous approach make therapy child-unfriendly. The transcutaneous technique, by contrast, is non-invasive and can be easily applied at home. It would benefit the patient and the parents if TTNS would form an equal alternative to PTNS therapy. Besides few reactions such as skin reactions from the adhesive electrodes, no serious adverse events have been reported in TENS therapy [3, 5].

Ramírez-García et al. conducted a prospective efficacy study on TTNS compared to PTNS therapy as treatments for adults with OAB. The authors could demonstrate the non-inferiority of TTNS in comparison to PTNS in decreasing daytime voiding frequency [6]. However, the non-inferiority of the transcutaneous technique over the percutaneous technique in a pediatric population has not been proven so far.

In the TaPaS trial—acronym for Transcutaneous tibial nerve stimulation, Percutaneous and Sham therapy—we will evaluate both the superiority of TTNS over sham therapy (part I) and the non-inferiority of TTNS compared to PTNS in children with idiopathic OAB (iOAB) (part II).

To our knowledge, only two sham-controlled RCTs have been published on the efficacy of TTNS in children with OAB. In the first study published by Boudaoud et al., 20 children with OAB were randomized into a TTNS and a sham group. The clinical results remained the same between both groups, underlining the potential placebo effect of any type of management in this population [7]. In the second study by Patidar et al. a significant benefit of TTNS over sham therapy in a study population of 40 children with OAB was seen [8]. Since the results of both trials are contradictive, we decided to set up a new similar RCT in order to be able to conduct part II.

### Objectives {7}

The objective of part I of the TaPaS trial is to evaluate the superiority of TTNS therapy over sham therapy. It is set-up as a small clinical trial in order to obtain own data, enabling us to conduct part II. The end objective of the TaPaS trial is to show the non-inferiority of TTNS over PTNS therapy.

### Trial design {8}

A prospective, single-center randomized controlled double-blinded trial is set up, comparing TTNS versus sham therapy. Participants are allocated on a 1:1 ratio to either the intervention arm, either or the control arm, according to a computer-generated list of random numbers. Superiority of TTNS over sham will be assessed.

## Methods: participants, interventions, and outcomes

### Study setting {9}

The recruitment of participants is carried out by pediatric urologists and pediatric nephrologists from the Department of Urology and at the Department of Pediatrics at the academic hospital of the Ghent University (East-Flanders, Belgium). Children attending a pediatric specialist (PS) for urgency incontinence or bedwetting are eligible for inclusion. The inclusion criteria are listed below.

At the initial visit, the child with lower urinary tract symptoms (LUTS) is subjected to a complete diagnostic work-up according to the standard of care [9]. Besides a baseline history-taking assessment, a few additional evaluations are performed: urine sample analysis, a bladder and kidneys ultrasound (US), and an uroflowmetry with an US guided post-void residual (PVR) measurement. A 3 days daytime bladder diary and a 7 nights night-time bladder diary are distributed [10]. Based on the completed bladder diaries and the baseline work-up, children are diagnosed at the second visit and subsequently screened for eligibility.

### Eligibility criteria {10}

Patients who meet the following criteria are eligible for inclusion:

- Diurnal urgency urinary incontinence and/or nocturnal enuresis (primary or secondary).
- Aged from 5 to 12 years old.
- Being treatment–naïve or only having been treated with urotherapy (see the “Intervention description {11a}” section for a detailed definition of urotherapy).
- Having a parent or guardian who is able to complete bladder diaries reliably.

Exclusion criteria are the following:

- Patients already treated with non-conservative therapies, i.e., any drugs with anticholinergic effect, transcutaneous electrical nerve stimulation (TENS), PTNS, or intradetrusor botulinum toxin injections.
- Patients with a neurogenic bladder dysfunction.
- Patients with dysfunctional voiding, diagnosed by the presence of a staccato-shaped curve on uroflowmetry at the moment of screening.
- Patients with nocturnal polyuria, registered on a 7 days night-time bladder diary. Nocturnal polyuria is defined by the International Children’s Continence Society (ICSS) as a nocturnal urine production exceeding 130% of the expected bladder capacity for age [10].
- Patients with behavior disorders like attention-deficit hyperactivity disorder.
- Patients with learning disabilities and/or lack of capacity, unable to comprehend urotherapy”

### Who will take informed consent? {26a}

If a child is eligible for inclusion, the child and parent(s) or guardian are verbally informed about the study by their PS and a written patient information sheet is distributed. If both parties are willing to participate, an informed consent for the parent and an informed consent adapted to minors (from 8 to 12 years old) are signed by the respective parties. Subsequently, an appointment for a first visit at the pediatric physiotherapist is made. A research assistant, also being a medical doctor, can function as an authorized surrogate to take informed consent.

Both the participant and investigator receive a signed copy of the consent form. Paper IC forms are stored and kept confidential in the archives of the data management unit. At all times, the IC form can be requested by the institutional data protection officer.

### Additional consent provisions for collection and use of participant data and biological specimens {26b}

There are no additional informed consents needed since no biological specimens are taken.

## Interventions

### Explanation for the choice of comparators {6b}

The results of the two only RCTs that have been conducted to compare TTNS and sham therapy in children are conflicting [7, 8]. Therefore, we chose to set up a new sham-controlled RCT.

### Intervention description {11a}

#### Intervention group

**Fig. 1:**
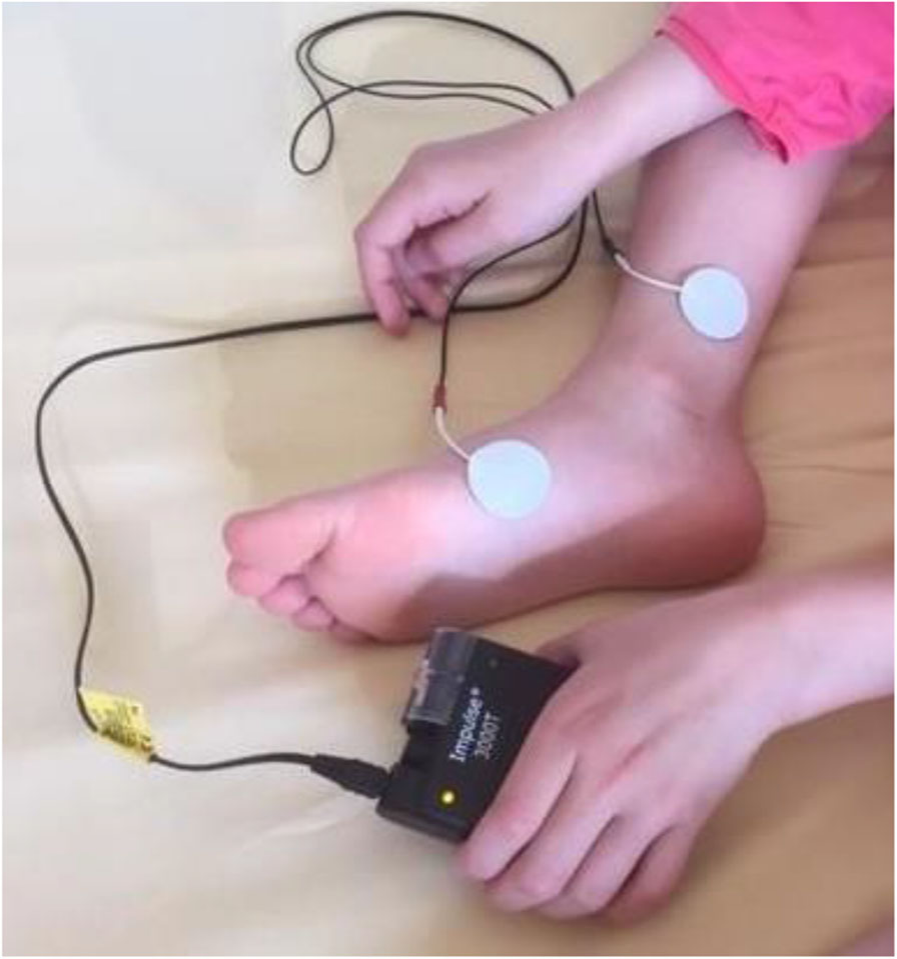
Application of the TENS device

Active neuromodulation treatment is delivered by the Transcutaneous Electrical Nerve Stimulation (TENS) device from BioMedical Life Systems, the Impulse 3000 T®. The physiotherapist places a round self-adhesive surface electrode (Ø3.2 cm) on the tibia, 2 cm superior from the medial malleolus (the negative electrode). The positive electrode is placed in the middle of the medial foot arch on the conduct of the posterior tibial nerve (see Fig. 1). Stimulation parameters are set on a fixed pulse width of 200 μS and a pulse frequency of 20 Hz. In literature, no consensus is reached on the ideal stimulation settings of TTNS [11]. Most studies report a pulse width of 200 μs [3]. We decided to choose the same frequency (20 Hz) as the previous study by Patidar et al. since significant results in this active treatment arm were seen [8].

During the therapy session, parents are instructed by the physiotherapist how to apply the surface electrodes themselves. By increasing the pulse intensity by turning the wheel, the TENS device will be switched on. The pulse intensity (in volt) is increased till a comfortable, painless sensation is felt. While increasing the pulse intensity, no motor responses may occur. During the following 30 min, in-office stimulation is given.

At the end of the session, the same device is given to the parents in order to apply ambulant therapy (AT). Parents are instructed to apply the TENS-device on the ankle of their child 1 h daily during the next 12 weeks (two times 6 weeks with 1 visit at the physiotherapist in between), without changing the fixed settings. The TENS device must always be placed on the same ankle. If habituation to the electrical impulse occurs, the pulse intensity should be set around the perception threshold. The intensity may be increased from 1 to 20 V, but stimulation may never be considered as painful. To conceal the allocation of treatment (effective treatment versus sham) for parents and child, they are told they cannot determine by sensation to which study group they belong. Parents who had previous experience with TENS devices are told they cannot compare this TENS therapy with their own since stimulation settings are each time different for every indication.

#### Sham group

The same TENS device, electrode positioning, pulse frequency (20 Hz) and pulse width (200 μs) as in the intervention group are applied for the sham group. Unlike the intervention arm, the pulse intensity button is set on position number “1,” which is the test button on which no electric current is delivered. Parent and child are told that subsensory stimulation will be delivered, not affecting the outcome as proven earlier in other types of neuromodulation trials [12]. In this way, we counter their worry that no sensation is felt. To discourage children or parents from exceeding number 1, they are told higher stimulations can affect the result of treatment in a negative way.

The same stimulation protocol as for the intervention group (1 h daily during 2 periods of 6 weeks) needs to be followed at home after the first visit. When switching the TENS-device on, they may not exceed number 1. This way, no current is supplied.

To check whether this method of blinding was successful, at the end of the trial, both parent and child are asked to indicate to which group they think they belonged.

#### Urotherapy

Besides neuromodulation, every participant in the trial will receive the same standard Urotherapy. Urotherapy can be defined as a bladder re-education or rehabilitation program aiming for the correction of filling and voiding difficulties [13]. It encompasses the following components [10]:

- Education on normal lower urinary tract (LUT) functioning and on how the LUT function of the child differs from normal.
- Lifestyle advices: counseling on drinking (1,5 L–2 L fluid intake daily) and voiding habits, diet, reduction of caffeine intake, proper stool management, etc.
- Behavioral modifications: sustaining regular voiding habits and adequate toilet positioning, avoiding holding maneuvers, etc.
- Regular follow-up and encouragement strategies: specifically for this trial, a follow-up visit at the physiotherapist will occur after 6 (the second visit) and 12 weeks (the third visit) of AT.
- Registration of symptoms, drinking, voiding, and stool habits through diaries.

Specifically for this trial, a 1-day bladder diary must be completed consistently every week as part of the urotherapy. Additionally, 3 times weekly, the participant must try to hold up urine for 5 min if the urge to urinate comes up. Gradually, the duration of delayed urinating should be trained to be increased. The so-called supervoids are part of urotherapy.

In the sixth week, a 7 day night-time bladder diary must be completed. After the second visit at the physiotherapist, the participant must continue the stimulation and urotherapy (plus completion of the diaries and supervoids) for another 6 weeks.

After the third visit, an observation period of 6 weeks is carried out during which no stimulation is given. Urotherapy however should be continued.

### Study schedule

**Fig. 2:**
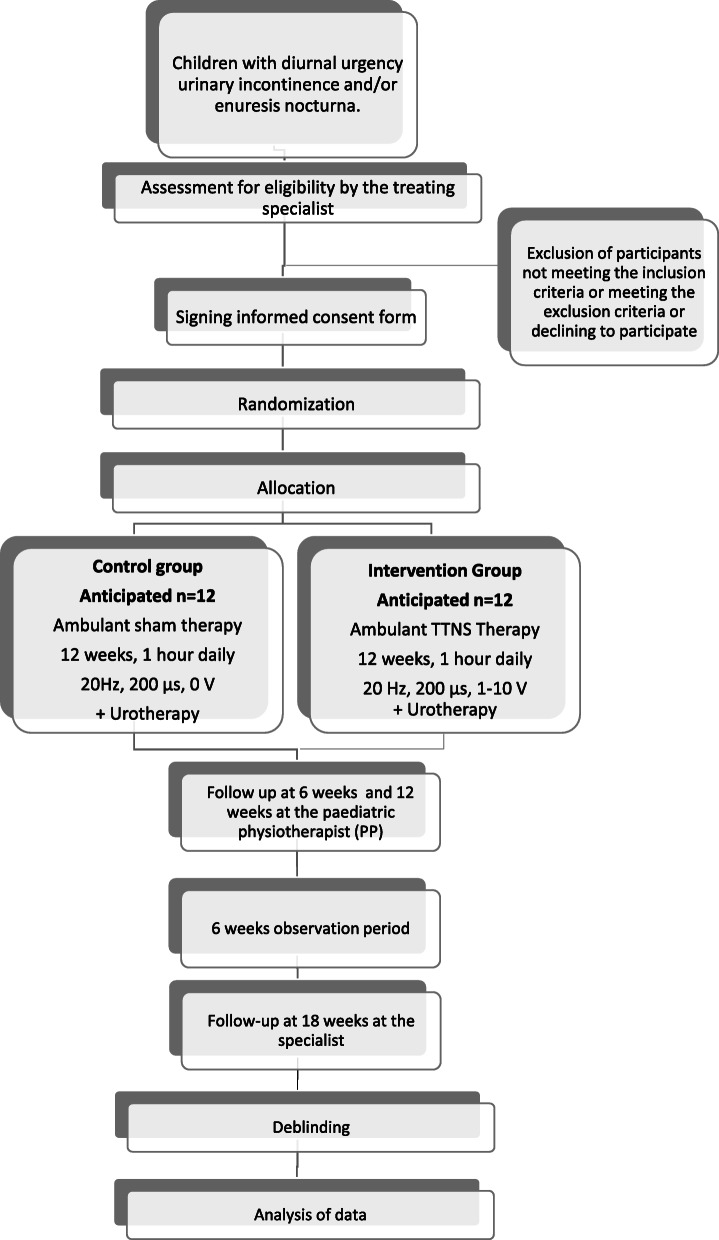
CONSORT flowchart of the TaPaS trial part I

For a structured overview of the study schedule, see Fig. 2 and Table 1. After enrolment, the participant is referred to the physiotherapist for a first visit (t_1_). Urotherapy and a first neuromodulation session are given. After home-therapy for 6 weeks, a second evaluation visit at the physiotherapist is planned (t_2_). Together with the parent and participant, the progression in symptoms is evaluated by review of the completed diaries. After the second 6-week period of home-therapy, a third follow-up visit at the physiotherapist is planned (t_3_). Primary outcomes are collected and delivered to the research assistant. At this point (at 12 weeks), the degree of clinical response to the treatment will be assessed. “No response” is defined as less than 50% increase in the average voided volume (AVV) compared to baseline, registered in the bladder diary. “Partial response” is predefined as 50–99% increase in the AVV, and “Complete response” as a 100% increase in AVV [10].

**Table 1:**
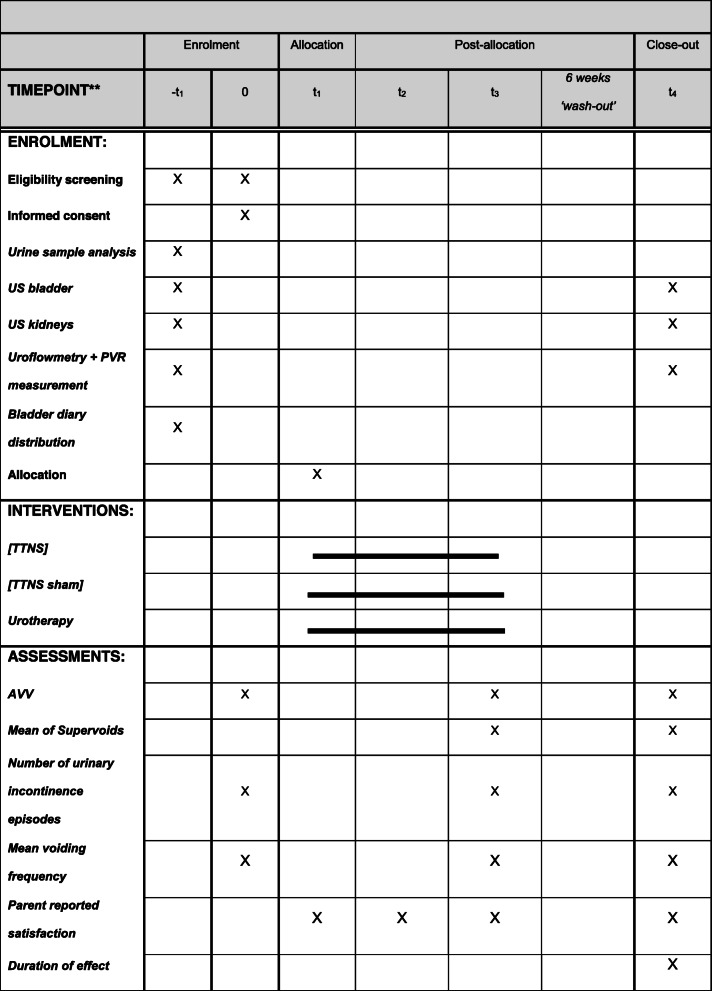
Schedule of enrolment, interventions and assessments

To evaluate the duration of clinical response, a third period of 6 weeks without any treatment (the observation period) is inserted. During this “wash-out” period, the same set of bladder diaries must be completed.

The study is closed at the end of the last follow-up visit at 18 weeks at the treating PS (t_4_). The PS will be briefed beforehand to which treatment arm the participant was allocated. The same assessment as during baseline will be repeated and the parent and participant will be unblinded during this follow-up visit.

For post-trial care, see item 30.

### Criteria for discontinuing or modifying allocated interventions {11b}

In case a dermal allergic reaction from the self-adhesive electrodes as an expected adverse event would occur, hypoallergenic electrodes will be provided to the participants, free of charge. On the participant’ request, the therapy can be discontinued at any time in the course of the study. No reason for this request should be given. In case symptoms would improve or worsen during therapy, no modifications to the stimulation protocol are allowed.

### Strategies to improve adherence to interventions {11c}

At the first study visit, extensive counseling of the importance of therapy compliance is given to the parents. Adherence to therapy will be stimulated by the required completion of bladder diaries and the active encouragement of the participant and parent by the physiotherapist during the 6-weekly follow-up visits. During the follow-up visits, the potential progression of symptoms will be actively evaluated by going through the completed diaries, together with the participant.

To check the adherence to the treatment protocol by the participant, registration of the performance and duration of daily TENS-application in a diary will be requested.

### Relevant concomitant care permitted or prohibited during the trial {11d}

During the trial, the following bladder medication may not be taken: Anticholinergic drugs and a β3 receptor agonist (i.e., mirabegron). Medication to stimulate bowel movement and treat constipation (such as laxative) are permitted. Another form of neuromodulation like sacral TENS is prohibited.

### Provisions for post-trial care {30}

The study is closed at 18 weeks. Participants who had received partial or complete clinical success at 12 weeks (independently from the allocated group) can either choose to stop any further treatment or receive further therapy along the standard of care (i.e., bed-wetting alarm if enuresis is still present, pharmacotherapy or percutaneous tibial nerve stimulation (PTNS)).

If participants belonged to the sham group and did not perceived clinical success, voluntary enrolment in part II of the TaPaS trial is possible, wherein TTNS will be compared to PTNS therapy. No financial support for post-trial care will be provided.

### Outcomes {12}

All outcomes will be assessed after 12 weeks of treatment and will be compared to baseline measures.

Outcomes will be reported both as:

- Median (IQR) absolute change from baseline − median (IQR) percentage change from baseline.

#### Primary outcome

* To show that TTNS therapy is superior to sham therapy in terms of average voided volume (AVV).

- The endpoint is the median change in baseline at 12 weeks in AVV, registered on the daytime bladder diary. The AVV is calculated by the sum of all voids during daytime (in ml) of 3 days in total divided by the frequency of voids during daytime over 3 days in total. We consider AVV as the most relevant variable to measure efficacy since it directly reflects the functional bladder capacity. Increasing the functional bladder capacity is the key of in OAB and/or nocturnal enuresis (in the absence of nocturnal polyuria) treatment. An increased bladder capacity should ultimately lead to a decrease in micturition frequency and in urinary incontinence episodes. The follow-up interval of 12 weeks was chosen since the effect of treatment can be expected after 12 weeks of treatment.

#### Secondary outcomes

##### The mean volume of supervoids

This is the median change in the mean volume of three “supervoids” between week 12 and week 1, registered on the 3 days daytime bladder diary. See the “Interventions” section for the definition of supervoids. Same as for the primary outcome, this outcome reflects the change in bladder capacity.

##### Number of urgency incontinence episodes during daytime and night-time

This is the median change of urinary incontinence episodes compared to baseline registered in a 3 days daytime bladder diary and night-time bladder diary (the average of 7 nights).

For parents and participants, this is the most relevant clinical outcome since it significantly affects daily activities mostly.

##### Mean voiding frequency during daytime

This is the median change in diurnal voiding frequency registered in the 3 days daytime bladder diary.

##### Parents reported satisfaction of urinary symptoms

Parents are asked to rate the following question: ‘How would you feel as a parent or guardian if your child’s bladder symptoms were to remain as they are on at this moment?” A score on a Likert-scale from 1 (extremely dissatisfied) to 7 (extremely satisfied) at baseline and after 12 weeks of treatment must be given.

The parent or guardian is the most trustworthy source to obtain a subjective assessment. Therefore, a parent reported instead of patient reported outcome is obtained.

##### Duration of effect

The duration of persistent partial or complete response during the observation period of 6 weeks, expressed in weeks. Same as during the treatment period, one diurnal bladder diary must be completed every week. This tells us more about the maintenance of clinical effect that is either received by active stimulation plus urotherapy or by urotherapy alone and a potential placebo effect.

### Participant timeline {13}

**Fig. 3:**
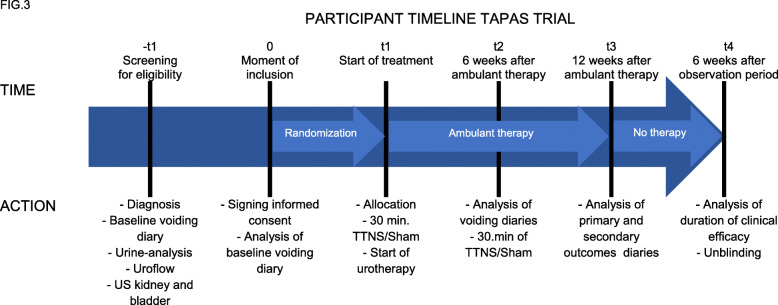
Participant timeline TaPaS trial

For a participant timeline see Fig. 3.

For a schedule of enrolment, interventions, and assessment, see Table 1.

### Sample size {14}

For the sample size calculation, an online software package was used:

Sealed Envelope Ltd. 2012. Power calculator for continuous outcome superiority trial. [Online] Available from: https://www.sealedenvelope.com/power/continuous-superiority.

The trial has a parallel superiority design. Based on the previous study by Patidar et al. [8], a mean difference of 20% in the primary outcome (i.e., percentage difference in AVV between baseline and after 12 weeks) between TTNS and sham can be expected, with an estimated standard deviation of 20. A sample size of 24 participants (12 in each treatment arm) is then required to achieve a power of 80% for two-sided testing at a 5% significance level.

### Recruitment {15}

To reach an adequate sample size, recruitment of participants is performed by different pediatric specialists from both the Urology and Pediatric Nephrology Department during the consultations. We chose to include patients who have not been treated yet with non-conservative therapies, among others to increase the number of potential participants. Additionally, treatment-naïve patients are easier to convince to participate in a sham-controlled trial than patients who have been unsuccessfully treated and want the guarantee of an effective treatment.

## Assignment of interventions: allocation

### Sequence generation {16a}

The randomization sequence list was computer-generated using the online platform “Sealed envelope” (available at: sealedenveloppe.com). Randomization is generated with a parallel 1:1 allocation of two treatment groups A and B, using random block sizes of 4. No stratification of participant characteristics was implemented.

Independently from the randomization sequence list, the physiotherapist defined randomly groups A and B (TTNS or sham).

Subsequently, the randomization sequence list was exempted for use to the physiotherapist.

### Concealment mechanism {16b}

The allocation sequence is not concealed for the physiotherapist, who assigns and implements the interventions. From the moment a participant is sent for a first visit to the physiotherapist, she allocates a number to the participant in order of appearance. The sequentially numbered participants will receive the treatment as indicated in the order of the randomization sequence list.

The research assistant, however, who analyzes all the data, is neither aware of the participant number, the group allocation per participant, nor of the identification of group A and B.

### Implementation {16c}

Participants will be enrolled by the PS or the research assistant. Afterwards, the allocation sequence is generated by coincidence. The participant schedules an appointment with the physiotherapist according to his or hers own preference in time and the availability of the physiotherapist. This sequence will determine the sequentially numbered list who is developed by the physiotherapist. Assignment of participants to interventions is also performed by the latter.

## Assignment of interventions: blinding

### Who will be blinded {17a}

The treatment is blinded for the patient, the parent, the treating PS, and the research assistant. The physiotherapist is the only unblinded party.

### Procedure for unblinding if needed {17b}

In case the participant and parent want to discontinue with the trial, the allocated intervention will be revealed. In no other circumstances, unblinding is allowed. The treating PS will be unblinded before the last follow-up visit at 18 weeks. The research assistant will be unblinded after all data has been entered into the database.

## Data collection and management

### Plans for assessment and collection of outcomes {18a}

Bladder diaries and the parents reported satisfaction of urinary symptoms questionnaire are distributed at the beginning of treatment on paper. The demographic data from each participant will be derived from the electronic medical record (EMR). All outcome data will be collected at the end of the 12 weeks treatment period and converted by the research assistant into a digital file. A standard frequency-volume chart with additional information of urinary incontinence episodes and urgency episodes will be used as bladder diary [10]. The parents’ reported satisfaction of urinary symptoms score is a non-validated 7 points Likert scale.

The following case report forms will be collected (see also Fig. 3 and Table 1):

-t1: First visit with the PS.

CRF with baseline patient characteristics: age, gender, presence of daytime urinary incontinence and/or enuresis nocturna, and comorbidities.

CRF with technical investigations: presence of urinary tract infection (yes/no), kidney ultrasound (US) (normal or abnormal), PVR on bladder US (ml), cystometric bladder capacity at uroflowmetry, and staccato pattern on uroflow suggestive for dysfunctional voiding (Y/N).

- 0: Second visit with the PS.

CRF with baseline daytime and nighttime diary parameters (volume intake, daytime diuresis, micturition frequency, AVV during the day, urgency episode (Y/N), number of urinary incontinence episodes, N of defecations, N of wet nights, nocturnal urine production, netto urine loss/night, presence of nocturnal polyuria), and parents’ reported satisfaction of urinary symptoms.

- t1: First visit with the PT.

CRF with patient number, allocation group.

- t2: Second visit with the PT, 6 weeks after ambulant treatment.

CRF with daytime and nighttime diary parameters after 6 weeks of treatment (same parameters as at visit 2 + averaged volume of 3 supervoids at 6 weeks) and parents reported satisfaction of urinary symptoms.

CRF with recorded (serious) adverse effects: the adverse event form.

*t3: Third and last visit with the PT, 12 weeks after ambulant therapy.

Same CRF’s as in t2.

CRF with treatment response (non-responder, partial responder, complete responder).

*t4: Third visit with the PT.

Same CRF’s as in t2.

CRF with the maintenance of treatment effect in partial and complete responders (in weeks).

### Plans to promote participant retention and complete follow-up {18b}

The short duration of the clinical trial (i.e., 18 weeks) should be the most important incentive to complete the whole trajectory. By inserting a follow-up visit in the middle of the study period, participants are promoted to continue the treatment protocol. Participants who discontinue treatment are no longer candidates to be included in part II of the TaPaS trial.

### Data management {19}

Data is entered manually in on an online firewall protected data registry on the password-encrypted server of the Ghent University. Subsequently, data will be stored in a central IBM SPSS database which is also protected by the institutional firewall. Only the trial coordinator has access to both data registries. To promote data quality, data will be controlled on impossible values by range checks.

### Confidentiality {27}

The participants’ data are pseudo-anonymized by assigning an unique code to every participant at the moment of inclusion to ensure confidentiality. Data will be stored during and after the trial in an online protected data registry and IBM SPPS database, only accessible by the trial coordinator and at the end of the study by the CI.

### Plans for collection, laboratory evaluation, and storage of biological specimens for genetic or molecular analysis in this trial/future use {33}

No laboratory examinations are performed; biological specimens are not to be collected.

### Statistical methods

#### Statistical methods for primary and secondary outcomes {20a}

Statistical analyses will be performed using SPSS version 25.0. Descriptive statistics of demographic characteristics will be presented by median and interquartile ranges (IQR) for continuous variables and frequencies and percentages for categorical variables. Since the small sample size, non-parametric tests will be chosen. In case a subject withdraws early, a new participant will replace them till the required sample size is reached. An ITT will be performed. If case data are missing, missing data will be imputed via multiple imputation.

- ° Intra-arm analyses (baseline vs. 12 weeks):
- Baseline and 12 weeks follow-up absolute values and absolute change and percentage change from baseline values will be presented as medians (IQR). Intra-arm analyses will be assessed using a two-sided Wilcoxon signed rank test.
- ° Inter-arm analyses (sham versus TTNS therapy): differences in absolute and percentage change from baseline between both groups will be reported as medians (IQR) and will be presented with a 95% confidence interval.

Inter-arm analyses will be assessed using a two-sided the Mann-Whitney *U* test

#### Interim analyses {21b}

No interim-analysis will be carried out.

#### Methods for additional analyses (e.g., subgroup analyses) {20b}

No subgroup analyses will be carried out.

Changes in fluid intake between baseline and after treatment will be statistically examined by a Wilcoxon signed rank test. In case a significant difference is noted, logistic regression analysis can be performed to examine whether change in fluid intake is an independent predictor for partial response or complete response to treatment or sham (> 50% increase in AVV at 12 months follow-up).

#### Methods in analysis to handle protocol non-adherence and any statistical methods to handle missing data {20c}

The non-adherence to the treatment protocol by the participant can be detected by controlling the diaries that report the daily duration of application of TENS-therapy. We are counting therefore on the participant’s reliability and honesty. In case non-adherence is noticed, besides the aforementioned intention-to-treat analysis, an additional per-protocol analysis will be carried out.

A missing value analysis will be performed. If data is missing (completely) at random, a multiple imputation model will be used, followed by a sensitivity analysis.

#### Plans to give access to the full protocol, participant level-data and statistical code {31c}

Participant data may be available upon request (lynn.ghijselings@ugent.be) after completion of the trial.

## Oversight and monitoring

### Composition of the coordinating center {5d}

The coordinating center is the Ghent University Hospital as an ERN eUROGEN accredited center. The trial management group is composed as follows:

- The CI: Prof. Dr. Anne-Françoise Spinoit.
- The trial coordinator: Dr. Lynn Ghijselings, i.e., the research assistant.
- The physiotherapist: Ms. Catherine Renson.
- Co-investigators: Dr. Lynn Ghijselings, Ms. Catherine Renson—an institutional biostatistician.
- An institutional data protection officer will protect every process of data management and will verify if every step in this process is according to current privacy regulations.
- The trial is monitored by the HIRUZ Clinical Trial Unit HIRUZ (Ghent University Hospital’s ‘Health, Innovation and Research’ department) Data Management Unit (DMU). They perform on-site and remote monitoring according to the International Council for Harmonization of Technical Requirements for Pharmaceuticals for Human Use – Good Clinical Practice (ICH-GCP) guidelines.

Since the small sample size of the trial, the decision was made not to have a trial steering committee.

### Composition of the data monitoring committee, its role and reporting structure {21a}

Since previous trials have been conducted with TTNS in children showing no serious adverse events [5], there is no formal appointed independent data monitoring committee. Any potential harm or risk to the patients will be assessed by a section of the HIRUZ clinical trial unit. This section has no conflict of interests in the trial or medical device company. In case of any risk to the patient’s safety, the chief investigator, sponsor of the trial and ethical board will be informed. Dependent from the severity of the AE, the decision to prematurely stop the clinical trial will be taken.

### Adverse event reporting and harms {22}

During each follow-up visit, the physiotherapist will query the participant for adverse events. In case adverse events occur during ambulant therapy, participants are asked to contact the physiotherapist. This information will be briefed to the CI, who will include all the details (time of occurrence, severity and attribution of the event—related or non-related to the therapy) on the adverse events form. AE will be quarterly reported to the Ethical Board or the Ghent University.

Adverse events will be treated along the standard of care.

Serious adverse events (SAE), also reported on the Adverse Event form, will be immediately (within 24 h) reported by the CI to the sponsor, the Ethical Board, and the FAGG (Federal Agency of Medicines and Health products). In case of any unexpected SEA of SUDA (serious adverse device effect), the study will be stopped. However, there are no SEA to be expected from TTNS, as previous studies in adults have proven its safety [3].

### Frequency and plans for auditing trial conduct {23}

An internal audit will be planned by the institutional clinical trial unit HIRUZ (clinical trials unit of Ghent University Hospital’s “Health, Innovation and Research” department.) This organization has no conflict of interests.

### Plans for communicating important protocol amendments to relevant parties (e.g., trial participants, ethical committees) {25}

In case important changes will be needed to be undertaken, an amendment on the original protocol will be communicated to the Institutional Review Board (IRB) and the co-investigators.

### Dissemination plans {31a}

In case the superiority of TTNS over sham therapy is proven, phase II of the TaPaS trial will be launched. Since showing the non-inferiority of TTNS compared to PTNS is the ultimate objective of the TaPaS trial, only results of part II will be communicated to a broader public. Trial results of part I will be submitted in the form of an original article for peer review in a not yet determined journal.

## Discussion

We do not expect there to be major practical or operational difficulties in the implementation of the trial. The biggest issue lies within the limited control on the adherence to treatment. Since the treatment is mainly home-based, there is little control on how strictly participants will have applied the TENS device daily. The requirement to complete regular bladder diaries and register the applied home–therapy does not guarantee full trial compliance and so compliance bias cannot be avoided.

### Trial status

The treatment protocol version 2 was approved by the Ethical Board of the Ghent University Hospital on 6 November 2018 (B670201837682). Recruitment has started in November 2018 and is still pending. Due to the COVID-19 pandemic, recruitment has been delayed. The expected study completion of recruitment of part I (TTNS vs. Sham) will be postponed to December 2021.

## Supplementary Information

**Additional file 1.**

## Abbreviations

AT: Ambulant therapy
AVV: Average voided volume: Sum of voided volumes per void during daytime over 3 days (in ml) divided by the sum of micturition frequencies during daytime over 3 days
CI: Chief investigator
CRF: Case report form
LUTS: Lower urinary tract symptoms
OAB: Overactive bladder syndrome
PTNS: Percutaneous tibial nerve stimulation
PP: Pediatric physiotherapist
PS: Pediatric specialist
PVR: Post-void residual volume
SAE: Serious adverse events
TENS: Transcutaneous electrical nerve stimulation
TTNS: Transcutaneous tibial nerve stimulation
US: Ultrasound
