# Supplementary material for: Clinical efficacy of transcutaneous tibial nerve stimulation (TTNS) versus sham therapy (part I) and TTNS versus percutaneous tibial nerve stimulation (PTNS) (part II) on the short term in children with the idiopathic overactive bladder syndrome: protocol for part I of the twofold double-blinded randomized controlled TaPaS trial
Source: Trials. 2021 Apr 2;22:247. doi: 10.1186/s13063-021-05117-8 (PMC8017511; doi:10.1186/s13063-021-05117-8)
Supplement: Supplementary file 1 — Additional file 1. [file 13063_2021_5117_MOESM1_ESM.docx]

Appendix

Information sheet for children (8-12 years)

**Title of the study:** Clinical efficacy of Transcutaneous Tibial Nerve Stimulation (TTNS) versus Sham therapy (Part I) and TTNS versus Percutaneous Tibial Nerve Stimulation (PTNS) (Part II) on the short term in children with the idiopathic overactive bladder syndrome: Protocol for Part I of the two-fold double blinded randomized controlled TaPaS trial.

Hello,

You are invited to take part in a clinical study. This is scientific research that is done to find out if neuromodulation with adhesive electrodes on the ankle has an effect on your bladder problems. You and your parents or legal representatives can decide together to participate in this study. If you do not understand something or if you want to know more, you can always ask questions to your parents and your doctor.

# Why am i invited to take part in this study?

We invite you to participate in a study to test if neurostimulation, using adhesive electrodes, has an effect on your bladder disease. If this is the case, we can test afterwards if this type of neuromodulation is as effective as neuromodulation using needles.

# WHAT TREATMENT WILL I receive IN THIS STUDY?

You will receive or real or sham nerve stimulation. Both therapies will be applied by adhesive stickers placed on the ankle. The physiotherapist will show you and your parent(s) how to apply the therapy during a first clinical visite. Afterwards, the therapy will be continued at home. It has to be applied 1 hour daily, during 12 weeks.

Apart from neuromodulation, the physiotherapist will also explain you how to keep your bladder healthy by training on drinking and voiding habits. This is called Urotherapy. Together with the daily nerve stimulation, you’ll have to train your bladder as well through active Urotherapy.

# Which tests will be done for the study?

We only ask you to fill in voiding diaries on a regular base. Your parents will help you to measure the amount of volume you drank and the volume of urine you voided or that you lost.

# WHAT HAPPENS IF YOU DO NOT WANT TO PARTICIPATE IN THE STUDY?

If you do not want to participate in the study or if your parents do not want you to participate in the study, you can say this to your doctor. Your doctor and the nurses will not mind if you don’t want to take part. The doctor will then look with you how your disease will be further treated according to the current standard.

You can always ask questions about the study at any time.

# To whom can you ask questions?

If you have any questions during your treatment or if there are any problems, you can always talk to your parents, the nurses and your doctor:

Name and phone number of your doctor:

…………………………………………………………………………………………………

…………………………………………………………………………………………………

**INFORMED CONSENT FORM FOR THE PARTICIPANTS (8-11 YEARS)**

**First and last name of the participant**:………………………………………………

Tick the box "YES" if you agree; if not, tick the box "NO".

| I have read the information letter and I received a copy.  I received an explanation about the study and I was able to ask all my questions. | | |
| --- | --- | --- |
| I understand that my participation in the study is voluntary and that I can stop the study at any time without having to give a reason. | | |
| I agree that: | **YES** | **NO** |
| 1. Information about me and my disease will be collected and will get a code. |  |  |
| 1. I will receive either real of sham nerve stimulation during the study. |  |  |
| 1. I will need to collect voiding diaries on a regular base. |  |  |

| Name and first name of the participant | | Date |
| --- | --- | --- |
| Name of the doctor* | Signature | Date |

2 copies must be completed. The original is kept by the investigator in the hospital for a period of 25 years, the copy is given to the participant.

*Tick by the doctor if agreed

| I declare that I have provided the necessary information regarding this study (the nature, the purpose, and the foreseeable effects) orally and a copy of the information document to the participant. |  |
| --- | --- |
| I confirm that no pressure has been exerted on the participant to allow him/her to participate in the study and I am prepared to answer any additional questions. |  |
